# Biodiesel Production from Alkali-Catalyzed Transesterification of *Tamarindus indica* Seed Oil and Optimization of Process Conditions

**DOI:** 10.3390/molecules27103230

**Published:** 2022-05-18

**Authors:** Noreen Sajjad, Raha Orfali, Shagufta Perveen, Sabiha Rehman, Aeysha Sultan, Taslim Akhtar, Arif Nazir, Gulzar Muhammad, Tahir Mehmood, Safina Ghaffar, Areej Al-Taweel, Muhammad I. Jilani, Munawar Iqbal

**Affiliations:** 1 Department of Chemistry, The University of Lahore, Lahore 53700, Pakistan; noreen.sajjad@chem.uol.edu.pk (N.S.); sabiharehman55@gmail.com (S.R.); anmalik77@gmail.com (A.N.); 2 Department of Pharmacognosy, Collage of Pharmacy, King Saud University, P.O. Box 2457, Ryiadh 11451, Saudi Arabia; rorfali@ksu.edu.sa (R.O.); sghafar.c@ksu.edu.sa (S.G.); amaltaweel@ksu.edu.sa (A.A.-T.); 3 Department of Chemistry, School of Computer, Mathematical and Natural Sciences, Morgan State University, Baltimore, MD 21251, USA; 4 Department of Chemistry, Division of Science and Technology, University of Education, Lahore 54000, Pakistan; ayesha.sultan@ue.edu.pk; 5 Department of Chemistry, Government Associate College (W), Mandi Bahauddin 50400, Pakistan; taslim_chemist@yahoo.com; 6 Department of Chemistry, Government College University Lahore, Lahore 53700, Pakistan; mgulzar@gcu.edu.pk; 7 Centre for Applied Molecular Biology (CAMB), University of the Punjab, Lahore 53700, Pakistan; tahir.camb@pu.edu.pk

**Keywords:** *Tamarindus indica*, biodiesel, transesterification, methyl esters

## Abstract

Biodiesel is considered a sustainable alternative to petro-diesel owing to several favorable characteristics. However, higher production costs, primarily due to the use of costly edible oils as raw materials, are a chief impediment to its pecuniary feasibility. Exploring non-edible oils as raw material for biodiesel is an attractive strategy that would address the economic constraints associated with biodiesel production. This research aims to optimize the reaction conditions for the production of biodiesel through an alkali-catalyzed transesterification of *Tamarindus indica* seed oil. The Taguchi method was applied to optimize performance parameters such as alcohol-to-oil molar ratio, catalyst amount, and reaction time. The fatty acid content of both oil and biodiesel was determined using gas chromatography. The optimized conditions of alcohol-to-oil molar ratio (6:1), catalyst (1.5% *w*/*w*), and reaction time 1 h afforded biodiesel with 93.5% yield. The most considerable contribution came from the molar ratio of alcohol to oil (75.9%) followed by the amount of catalyst (20.7%). In another case, alcohol to oil molar ratio (9:1), catalyst (1.5% *w*/*w*) and reaction time 1.5 h afforded biodiesel 82.5% yield. The fuel properties of *Tamarindus indica* methyl esters produced under ideal conditions were within ASTM D6751 biodiesel specified limits. Findings of the study indicate that *Tamarindus indica* may be chosen as a prospective and viable option for large-scale production of biodiesel, making it a substitute for petro-diesel.

## 1. Introduction

Energy has long been regarded as one of the most critical aspects of daily life. Humans depend primarily on nonrenewable energy sources such as coal, petroleum, and natural gas. However, burning of fossil fuels causes environmental pollution. The only realistic way to address the energy crisis is to find clean and climate-friendly alternative energy sources. Because of the global energy crisis, biodiesel has gained greater attention as an alternative energy source. Biodiesel is a kind of fuel produced via transesterification of oils derived from animals or plants yielding ethyl, methyl, or propyl ester [1,2,3,4,5,6,7].

The use of discarded materials such as tallow, animal fat, and cooking oil is sustainable and a more environmental friendly method of energy production. The consumption of organic waste not only produces energy but also serves as a means of solid waste management [8,9]. Biodiesel’s benefits include its liquid nature, renewability, environmental friendliness, higher combustion efficiency, cetane number, biodegradability, higher flash point, and lubricity [10].

Biodiesel can be produced by different methods such as micro-emulsion, thermal cracking, direct use and blending, and transesterification. A micro-emulsion approach produces fuel with lower viscosity and cetane number. Biodiesel produced from thermal cracking is similar to gasoline obtained from petroleum, but it is more expensive. A direct use and blending approach has the advantage of producing more portable fuel due to its liquid nature, but the fuel has a higher viscosity [11,12].

Transesterification is the catalyzed reaction of oil or fat with alcohol to yield an ester and glycerol. Biodiesel production using transesterification is an economical and time-saving method and produces fuel with higher cetane number and improved performance. Transesterification is a popular method for converting vegetable oils such as *Eriobotrya japonica* seed oil [13] and non-vegetable oils such as *Tamarindus indica* (*T. indica*) seed oil into biodiesel. Tamarind belongs to the *Fabaceae* (*Leguminosae*) family of dicotyledonous plants. The trees are ideal for low input because of their ability to grow in poor soils and endure long periods of drought [14,15,16].

Currently, an equivalent of nearly 11 billion tons of fossil fuel is being consumed around the globe. Crude oil reserves are decreasing at a pace of 4 billion tons per year and will be depleted by 2052 at this pace [17]. As a result, a home-derived alternate fuel source is urgently required. Biofuel is ready to meet the demand when the present oil field outputs are declining and new fields are not yet operational. Biofuels will go a long way toward filling the gap between limited fuel sources and rising demand throughout the world, which is probably certain to increase in the coming years [18].

As far as the feedstock is concerned, *T. indica* seed oil has not been studied in detail previously despite its vast occurrence in Asian regions such as Pakistan. Lack of detailed previous studies, appreciable oil content, vast occurrence, and nonexistent competitive uses make *T. indica* seed oil a novel and remarkable potential candidate for study as a renewable feedstock for the production of biodiesel. The main goal of this investigation is to use non-edible *T. indica seed oil* as an inexpensive and sustainable potential feedstock to make biodiesel using a base-catalyzed transesterification protocol and optimize the main factors affecting *T. indica* seed oil transesterification.

## 2. Materials and Methods

*T. indica* seeds were procured from local market in Lahore, Pakistan. A Soxhlet assembly (Zhengzhou Wollen Instrument Equipment, Henan, China) and heating mantle were used to extract oil. *T. indica* seeds were dried in sunlight for a few days to remove moisture content. Dried seeds were ground to fine powder in an electric grinder. The powdered sample was preserved in air-tight jar for further use.

### 2.1. Extraction and Transesterification of Tamarindus Indica Seed Oil

To extract their moisture content, *T. indica* seeds were dried in sunlight for a few days. The dried seeds were ground in an electric grinder and transferred to a soxhlet extractor (Zhengzhou Wollen Instrument Equipment, Henan, China) with a 250 mL round-bottom flask and *n*-hexane as the extraction solvent. Using a heating mantle set at 50 °C, the extraction was completed in about 4 h. At 46 °C under vacuum, the solvent was extracted from the oil using a rotary evaporator (Büchi Rotavapor R-215, New Castle, DE, USA). The concentrated oil thus obtained was dried with anhydrous Na_2_SO_4_ and filtered later.

The dried oil was subjected to transesterification in a round-bottom glass reactor (250 mL) supported with a condenser, thermostat, and sampling outlet. The seed oil (100 g) and methoxide solution (prepared by mixing predetermined amounts of catalyst NaOH wt. % of oil in methanol-to-oil ratio in moles) was stirred in a reactor at 500 rpm and at a desired temperature. After the reaction had been carried out for a given time, the reactor contents were transferred to a separating funnel. After 12 h, heavier glycerol layer sank to the bottom, while the lighter biodiesel fraction rises above it. After glycerol separation, the biodiesel was washed three times with warm purified water to remove impurities such as traces of glycerol and catalyst and any excess methanol that may have been present. The washed biodiesel was then dried through anhydrous Na_2_SO_4_ [18]. The percentage output of biodiesel was estimated via the formula given below (Equation (1)):

(1) $$\%\;\mathrm{Biodiesel}=\frac{\mathrm{Biodiesel}\;\mathrm{weight}}{\mathrm{Weight}\;\mathrm{of}\;\mathrm{oil}}\times 100$$

### 2.2. Gas Chromatography

**Figure 1:**
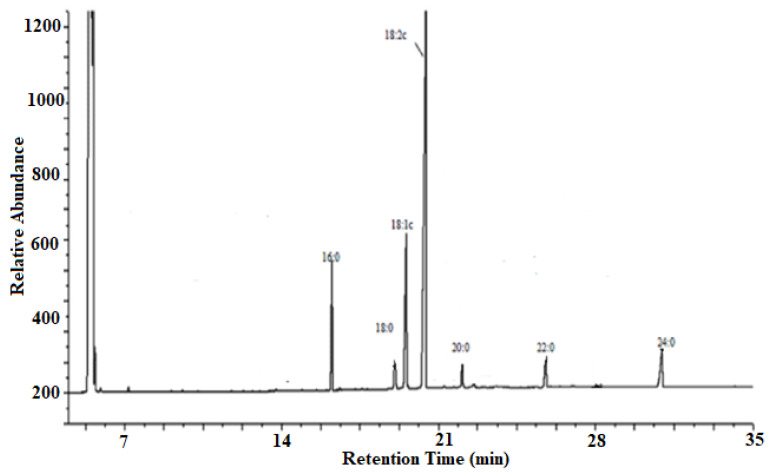
Representative GC FID Chromatogram of *Tamarindus indica* seed oil exhibiting the molecular composition.

The composition of fatty acid of biodiesel and *T. indica* seed oil was analyzed (Figure 1) using gas chromatograph (Agilent 7890A, Agilent Technologies, Santa Clara, CA, USA) fitted with a DB-23 column with a thickness of film of about 0.250 m, an inner diameter of 0.250 mm, and a length of 30 m. Flow rate of carrier gas (nitrogen) was adjusted to 2.80 mL·min^−1^. Other chromatographic conditions include a 190 °C initial oven temperature, 2 °C/min ramp rate, 220 °C inlet temperature, 220 °C final temperature, 300 °C detector temperature, and 0.2 μL injected sample volume.

### 2.3. Experimental Design through Taguchi Method/Orthogonal Array

Design of experiments (DOE) is a standard statistical method for designing processes and products and for resolving production issues. Other statistical approaches such as response surface model (RSM) are also being used for the optimization of process parameters. However, Taguchi method was preferred for the present study due to its ease of utilization, as it involves only a few most relevant parameters and requires a minimal number of experiments to reach the same conclusion, in comparison to the aforementioned methods, saving time as well as energy. The Taguchi method provides a standard version of the design of the experiment that allows us to implement the method to improve product design and investigate issues related to production [18]. The Taguchi DOE technique uses orthogonal array to optimize impact of various parameters on the level and process how they could be differentiated. This approach is notable because it does not consider all the possible parameter combinations; instead, only a few pairs are considered. As a result, a minimum number of experiments are needed to collect the data to evaluate the factor(s) affecting the product’s quality/yield. The orthogonal array (OA) allows for finalizing the necessary experiments and their settings quantitatively. The number of specifications and their levels of variance for every parameter are used to determine the OA form. The minimum number of experiments (*N*) can be computed by multiplying the levels (*L*) with design and control parameters (*P*) using the following Equation (2):

(2) N=(L−1)P+1

### 2.4. Control Parameters and Levels Selection

The reaction time, reaction type, and quantity of alcohol (ratio of alcohol to vegetable oil), type and amount of catalyst, mixing intensity or agitation speed (rpm), reaction time, purity level in oil, and moisture content of oil affect biodiesel yield through transesterification. Only three of the most significant variables were selected, and three different levels were considered in this study, namely *L* = 3 and *P* = 3, as shown in Table 1. The figures led to the L9 OA design, in which three parameters were investigated at three levels using just nine experiments, as indicated in Table 2. Every experiment was performed thrice to reduce the chance of error.

### 2.5. The Analysis of Variance and Signal-to-Noise Ratio (SNR)

The use of loss function was suggested in the Taguchi method to measure the deviation between desired value and experimental output properties. The value of the loss function is transformed further into the signal-to-noise ratio (SNR). The SNR is essentially the log function of the probable result that would serve as the optimization process goal. SNR is then used to measure the extent of deviation of the function quality from the predicted values. Depending on the problem’s goals, there are three types of SNRs in Taguchi method: smaller-the-better (STB) for problems with minimization, larger-the-better (LTB) for problems with maximization, and nominal-the-best (NTB) for problems with normalization.

Equations (3)–(5) to calculate SNR for STB, LTB, and NTB models are shown below:

(3) $$\mathrm{smaller}\;\mathrm{the}\;\mathrm{better}\;-\mathrm{SNRi}=-10\log\left(\overset{n}{\underset{j=1}{\sum }}\;\frac{y^{2}j}{n}\right)$$

(4) $$\mathrm{larger}\;\mathrm{the}\;\mathrm{better}\;-\mathrm{SNRi}=-10\log\frac{1}{n}\left(\overset{n}{\underset{j=1}{\sum }}\;\frac{1}{y^{2}j}\right)$$

(5) $$\mathrm{Nominal}\;\mathrm{the}\;\mathrm{best}\;–\mathrm{SNRi}=10\log\left(\frac{{\;\bar{y}}_{i}^{2}}{S_{i}^{2}}\right)$$

where $\bar{y}$_i_ represents the mean response value, S_i_^2^ is variance, and i, *j*, and *n* stand for experiment number, trial number, and the number of experiments, respectively.

For the determination of optimum parameter combinations, SNR-based evaluation of experimental data is usually performed. Since the current project aims for the highest biodiesel yield, out of three SNR quality features, a larger-the-better (LTB) model was applied. The optimum design/control parameter level would therefore be the highest SNR.

The optimum level of every factor/parameter can be achieved with the aid of SNR analysis. The optimum set of parameters leading to the maximum yield of the desired product is not yet possible to evaluate, because the extent of each parameter to performance is unknown. These contributions can, however, be identified by carrying out an analysis of variance of the response data. For this reason, it is essential to calculate the sum of squares. For the percentage (%) contribution estimation, the following equations are used (Equation (6)):

(6) $$\mathrm{Percentage}\;\mathrm{contribution}\;\mathrm{of}\;a\;\mathrm{factor}=\frac{{\mathrm{SS}}_{f}}{{\mathrm{SS}}_{t}}\times 100$$

The fth factor represents the SS_f_ sum of squares, while all parameters have the SS_t_ sum of squares.

### 2.6. Characterization of Seed Oil and Biodiesel

The oil content (%) was calculated from the weight of the oil in the seeds. Different physicochemical features of *T. indica* seeds oil were discovered by test methods (Table 3).

In a beaker, a fat sample of 1.0 g was taken and dissolved into 10.0 mL of alcohol solvent. In addition, 20 mL of ethanolic KOH 0.2 M standard was added to the fat solvent solution and labeled the sample. Without a fat sample, the process proceeded to synthesis for blank sample. Then, both samples were attached to the reflux condenser and heated for about 30 min to the boiling point of the water. The sample and blank were then allowed to reach 25 °C temperature. The phenolphthalein indicator of 2–3 drops was added in sample and blank and titrated against 0.2 hydrochloric acid normality. Saponification value was calculated using the equation below (Equation (7)):

(7) $$\mathrm{Saponification}\;\mathrm{Value}=\frac{M_{w}\times N\times \left(V_{blank}\times V_{test}\right)}{W_{s}}$$

where *M_w_* represents KOH molecular weight, g/mol; *V_Blank_* represents HCl volume for blank, in mL; *V_test_* represents HCl volume for sample, in mL; N represents KOH normality, mol/mL; and *W_S_* represents sample weight, in grams.

The specific gravity was measured by taking the difference in weights using specific gravity bottles. The sample was taken into the flow time viscometer (Cannon Fenske Opaque, Glass capillary viscometer, State College, PA, USA). The viscometer was kept in a metal holder and placed in a water bath at 40 °C for about 10 min to enable the sample to reach the bath temperature. The suction force was then applied to the thinner arm to raise the sample up to the mark. The time for free flow of the sample from the upper to lower marks was used to calculate the viscosity. A few drops of oil were placed on the refractometer’s glass slide, and the refractive index value was recorded.

Iodine value was calculated with 0.2 g of the oil sample dissolved in 10 mL of CCl_4_, followed by addition of 12.5 mL of Dam’s reagent. The flask was placed in darkness for 2 h. A total of 10 mL of KI (10%) and 62.5 mL of distilled water were added. The solution was titrated against 0.1 M sodium thiosulphate solution until the disappearance of yellow color. A total of 1% starch indicator was added dropwise, and titration was continued until disappearance of blue color by adding sodium thiosulfate dropwise. Iodine value (IV) is determined by the expression shown below (Equation (8)):

(8) $$\mathrm{IV}=\frac{12.69\times C\;\left(V_{1}-V_{2}\right)}{M}$$

where C = Na_2_S_2_O_3_ concentration, *V*_1_ = volume of Na_2_S_2_O_3_ for blank, *V*_2_ = volume of Na_2_S_2_O_3_ used for sample, and *M* is the sample mass.

Acid value was determined with 10 mL of ethanol mixed with 5 g of oil in titration flask, and phenolphthalein indicator was added dropwise. The solution mixture was titrated with 0.1 M KOH from colorless to dark pink color, and the volume of 0.1 M KOH (V_o_) was noted.

Cloud point (CP) and pour point (PP) are two low-temperature flow properties of biodiesel that must be considered when running compression-ignition engines in a moderate-temperature setting during the winter months. CP indicates the temperature at which a fuel becomes cloudy, indicating the formation of wax crystals, and PP exhibits the temperature below which the fuel ceases to flow.

Flash point is the lowest temperature at which a chemical can vaporize and form a flammable mixture. A lower flash point shows higher flammability. The sample of biodiesel is heated and the vapor is collected inside the cup at the time when vapor is noticed and the temperature measured is sufficient to ignite the flash light.

The cetane number (CN) of biodiesel is generally higher than that of conventional diesel. One of the most important indicators of diesel fuel efficiency is the CN. This refers to the time it takes for a fuel to ignite after being injected into the combustion chamber. The CN is a measure of the efficiency with which diesel fuel is ignited, with a high CN indicating a short delay in ignition. Biodiesel made from animal fats has a higher CN than biodiesel made from vegetable oils.

## 3. Results and Discussion

### 3.1. Physicochemical Properties

The *n*-hexane extracted seed oil was assessed for its potential as a feedstock for biodiesel production. In this respect, various physicochemical properties and fatty acid profile of oil were determined. Based on its fatty acid content and properties, base-catalyzed transesterification was implemented to prepare fatty acid methyl esters using sodium hydroxide as a catalyst. The physicochemical properties of seed oil can be seen in Table 3.

The seeds were dried in an oven for 3 h to reduce the humidity, which was up to 8%. The oil content from dried seeds was 16% (*w*/*w*), excellent for a biodiesel feedstock. The FFA content (%) of the oil (1.97%) indicates that it is possible to use base-catalyzed transesterification without pretreatment. The kinematic viscosity was 29.5 mm^2^/s, in agreement with the composition of the fatty acids (Table 4).

The seed oil consisted of unsaturated fatty acids such as oleic acid (14.52%, C_18:1_) and linoleic acid (61.51%, C_18:2_) and saturated fatty acids such as palmitic acid (9.90%, C_16:0_), stearic acid (2.22%, C_18:0_), Eicosanoic acid (1.50%, C_20:0_), Behenic acid (3.90%, C_22:0_), and Tetracosanoic acid (6.45%, C_24:0_). Therefore, unsaturated fatty acids contribute more (76.03%) than the saturated fatty acids (23.97%).

**Figure 2:**
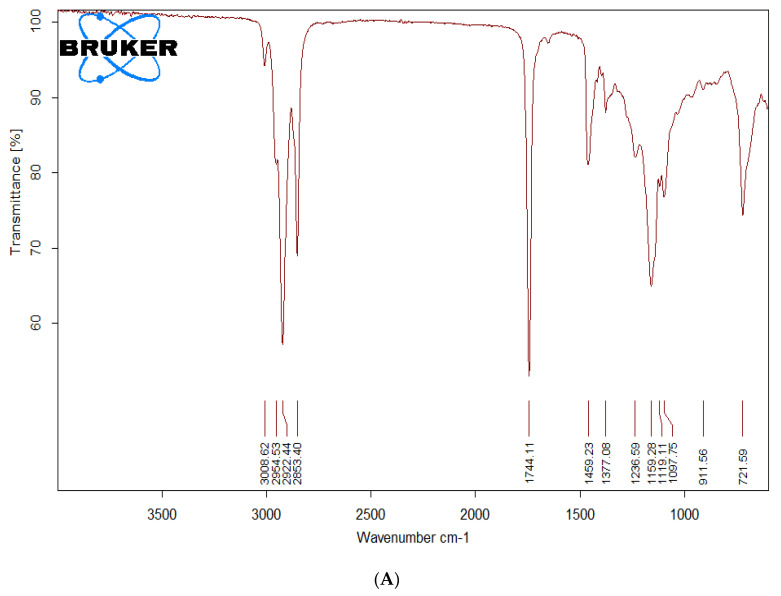

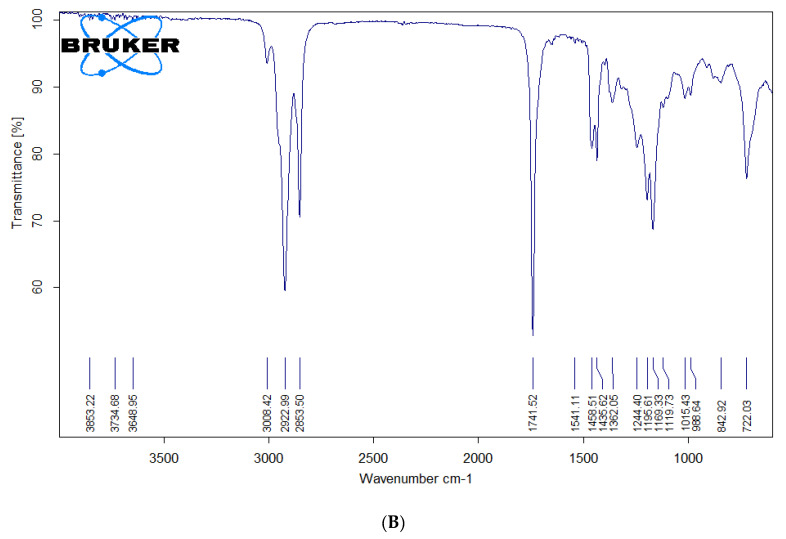
FTIR spectra of *Tamarindus indica* (**A**) seed oil and (**B**) methyl ester (biodiesel) representing different functionalities.

The formation of biodiesel was inferred from FTIR spectroscopy by comparison of spectra of precursor as well as transesterified product. As the precursor and final product are both esters differing only in the alkoxy moiety, it is quite rational to expect FTIR spectra of both the samples to be quite similar. However, there are few minor differences which can be used to differentiate the two. The region of C=O stretching typical of esters (1800–1700 cm^−1^) is quite similar in precursor oil and transesterified product. The main spectral region that allows for chemical discrimination between precursor oil and the corresponding methyl ester is specifically the finger print region. The asymmetric stretching of –CH_3_ appearing typically at 1446 cm^−1^ is characteristic for the methyl ester (i.e., biodiesel) and is absent in the FTIR spectra of precursor oil. The O–CH_2_ stretching of glycerol group (mono-, di- and triglycerides) appearing at 1377 cm^−1^ is only present in oil spectrum and is expected to be absent in methyl ester derivative of oil. The peak around 1196 cm^−1^ corresponds to O–CH_3_ stretching, which is typical of biodiesel, and is absent in precursor oil. The asymmetric axial stretching of HO–CH_2_− moiety appears in the range of 1075–1100 cm^−1^. These peaks are present only in the spectrum of oil and are absent in that of biodiesel. The specific fingerprint region is between 400 cm^−1^ and 1500 cm^−1^ in IR spectrum [19]. Figure 2 represents the FTIR spectra of the precursor oil (A) and the biodiesel (B) prepared by transesterification of the *T. indica* seed oil.

### 3.2. Optimal Process Parameters

Table 5 shows the experimental yields of *T. indica* methyl esters (TIMEs) from transesterification of seed oil, which were calculated using the L_9_ orthogonal array. The table also includes the computed SNR and the average mean SNR values. As reported previously, the current study’s target necessitated adopting the ‘larger the better’ SNR model. Experiment 6 had the highest mean yield (93.17%) and SNR (39.41). On the other hand, Experiment 1 recorded the lowest mean yield (61.33%), with an SNR of (35.75). However, the set of parameters corresponding to maximum yield may not be the optimum set of parameters.

Table 6 shows the SNR_L_ (level mean SNRs) values for each experimental variable at each level listed. For example, the SNR_L_ for parameter A at level 1 has been calculated as ‘36.51’ using SNR values from Experiments No. 1, 2, and 3. At level 2, it has been calculated as ‘38.88’ using SNR values from Experiments No. 4, 5, and 6, and so on. The SNR_L_ values for each parameter at various levels demonstrate its impact on TIMEs yield. The higher the effect of a particular parameter at a given level, the higher the value of SNR_L_. The maximum value of SNR_L_, which is directly related to the maximum yield of TIMEs, corresponds to the optimum level of every parameter. The optimum levels for parameters A, B, and C were 1, 2, and 3, respectively, corresponding to the methanol-to-oil ratio of 6:1, an amount of catalyst 1.5% (wt./wt.), and a time of reaction of 2 h.

### 3.3. Analysis of Variance (ANOVA)

The highest percentage contribution is furnished by the molar ratio of alcohol to oil, i.e., 75.9%, which is in agreement with findings of Akhtar et al. [13], for the production of biodiesel from cantaloupe seed oil, wherein the contribution of this experimental variable was even higher. Similarly, a small contribution was furnished by the reaction time which again complies with the above mentioned study. Furthermore, these percentage contributions are in correspondence with the variation in their respective SNRL values [20]. The computed sum of squares (SS) for each parameter and the paramaters’ percentage contributions are shown in Table 7. These findings aid in determining the most critical parameter with the most significant impact on the TIMEs yield. The ‘molar ratio of alcohol’ used to transform oil to methyl esters has the greatest effect (75.9%), followed by catalyst amount in relation to oil (20.7%) and reaction time (3.37%). This could be linked to minor variations in SNR_L_ values for each parameter at three levels. In other words, the % contribution of a parameter (∆SNR) to the mean yield of the final product is directly determined by the difference between the minimum and maximum SNR_L_ values for that parameter. These parameters can be ranked using ∆SNR calculations, with the highest rank going to the parameter with the highest value of ∆SNR. According to the data in Table 6 and Table 7, the ‘alcohol to oil molar ratio’ ranks first, followed by the catalyst amount and reaction time. In three replicate trials, the optimal levels of all parameters were used to assess the percentage yield of TIMEs. The average biodiesel yield was 93.5%, similar to the result obtained in Experiment No. 6. As for optimization of biodiesel production using the Taguchi method, the amount of catalyst, molar ratio of alcohol to oil, and reaction time and temperature of reaction were influencing parameters [20].

### 3.4. Fuel Properties of Methyl Esters

Table 8 shows the different fuel characteristics/properties of biodiesel obtained from the transesterification of seed oil. The kinematic viscosity was determined to be 5.4 mm^2^/s, critical in the spraying of fuel and the formulation of mixtures and combustion procedure. However, high kinematic viscosity interferes with the injection process, causing improper atomization of the fuel. Thus, it should be kept within limits defined in biodiesel standards such as ASTM D6751. The flash point (FP) was discovered to be 180 °C, which is an essential feature of fuel in terms of transportation and storage protection. The fuel volatility is also linked to flash point, a significant factor in starting and warming an engine. Low fuel volatility combined with high viscosity causes weak engine start-up (cold), ignition delay, and misfire [21]. The pour point and cloud point of a liquid fuel were −2 and 5 °C, which correspondingly determine a fuel’s cold-weather consistency.

An acid value greater than 3% causes various operational issues such as pump corrosion and deposit formation. The acid value of the prepared biodiesel was 0.31 mg KOH/g, which is within acceptable limits. CN measures fuel ignition delay, a period between fuel injection and combustion. The ignition delay decreases with increasing cetane number, allowing the main combustion process to extend (diffusion-controlled combustion). The CN of prepared biodiesel is 47, which is well within acceptable limits. If the CN of fuel is greater than 65, it can ignite in a short time and at a great distance from the injector, allowing it to overheat, resulting in cooked particles and blocking of the injector nozzle. The Cu-strip corrosion, the one remaining characteristic, was also found to be within defined limits. Thus, *T. indica* seed oil can be used as a possible feedstock to synthesize biodiesel, since all of the fuel properties are within ASTM D6751 limits (Table 8).

Biodiesel has some added benefits when it is compared with traditional diesel fuels because of its lesser pollution, lower toxicity, eco-friendliness, and renewability attributes. Through the process of transesterification, biodiesel could be synthesized from different edible and non-edible sources. Non-edible available resources are normally consumed to prepare biodiesel because of their lack of dependency on the food chain and low cost. Non-edible sources include non-edible vegetable oil, algal oil, animal waste oil, and cooking waste oil. The production mechanism depends upon various factors such as time and temperature of reaction mixture, alcohol-to-oil molar ratio, catalyst type, and catalyst concentration. Through use of alternate technologies, different suitable types of catalyst, and suitable feedstock, the cost of biodiesel could be reduced from an economic perspective. Furthermore, cost could be reduced through some low-cost alternate raw materials, selling of by-products, operational labor cost, optimum operation conditions, certain types of catalysts, and mechanism of reaction. One of the major by-products which is produced during its reaction is crude glycerol, which has yields ranging from 8.0% to 10.1%. This crude glycerol could be used to prepare hydrogen, biopolymers, and ethanol or serve as an additive for fuel via gasification and pyrolysis processes. However, this work is more concerned with transesterification for non-edible reserves of biodiesel preparation along with economical aspects, by-product application, and fuel characteristics. Lastly, process optimum conditions along with economic parameters for biodiesel production should be evaluated as important parameters in order to achieve economic sustainability for biodiesel production [22].

Various methods for biodiesel production through different feedstocks have been employed in recent past. Increase in the price of petroleum-based fuels and depletion of energy reservoirs has led to the exploitation of nonrenewable resources. Therefore, there is a certain need to look for sustainable and suitable alternatives to traditional fuels. The major attributes for substituted fuel should be renewability, ready availability, and lesser dependency on limited resources, which could result in lesser pollution or no pollution at all. Biodiesel has attracted interest because of its eco-friendly and non-toxic properties. Nano-catalyst technology has also been used in recent years for the synthesis of biodiesel because of its various positive attributes such as re-usability, large surface area, and increased activity [21].

It is reported that biodiesel prepared from the indigenous plant *Salvadora persica L*. seed oil meets the international standards for biodiesel (ASTM D6751) and a one-step transesterification procedure is sufficient for the preparation of biodiesel. The yield of biodiesel was 1.57 g/5 g (31.40 percent by weight), and in-situ transesterification ester content conversion was 97.70%. Density of the biodiesel produced was 0.893 g/mL, the kinematic viscosity was 5.51 mm^2^/s, 210 °C was the flash point, CN was 61, and sulfur content was 0.0844%. At 595 °C, full oxidation of biodiesel resulted in a 97.0% weight loss, according to thermal analysis [23,24].

Waste-oil-based biodiesel is a better approach to preparing economical biodiesel, but a problem that may arise is that the process of transesterification may be hindered by the presence of much higher quantity of the free fatty acids (FFA). This process involves the consumption of wasted cooking oil in order to prepare good yield of biodiesel. Higher percentages of FFA were reported through acid value (5.49 mg KOH/gm) testing of waste cooking oil. Esterification processes were utilized through various acid-based catalysts such as sulfuric acid, hydrochloric acids and phosphoric acid. Among all, sulfuric-acid-based catalyst was proved to be more effective, because its FFA value was reduced to 88.78% at 59 °C, with oil-to-methanol molar ratio 2.5:1. The process of transesterification was carried out in the presence of alkali-catalyst-like potassium hydroxide, while output of fatty acid methyl ester was a maximum of 94% in the presence of 1.1% catalyst at 510 °C. Biodiesel produced from these reactions was evaluated through different tests such as specific gravity, acid value, cloud point, calorific point, iodine value, break test, CN, saponification value, and pour point. Further biodiesel testing was performed through gas chromatography. Waste-cooking-oil-based biodiesel was best synthesized through an alkali-catalyst-based transesterification process. Through data analysis, it was proved that waste cooking oil could be used as an effective resource for biodiesel production along with lessening environmental pollution for society [1,18,19].

The sustainability of the supply of fuel dependent on petroleum has received wide attention due to increased use in different industries and petroleum resource depletion. In the global community, market values for crude oil are volatile and uncertain. Additionally, there are also environmental issues rising due to emissions of toxic contaminants and greenhouse gases. Accordingly, it is important to use renewable energy sources, including biodiesel. Biodiesel is primarily created from non-limited resources of nature by a method of transesterification. This offers different benefits; for example, it is nontoxic, biodegradable, and eco-friendly over petro-diesel; the emissions it creates, apart from being healthy, have minimal sulfur and aromatic content [25].

A daunting challenge is to use sustainable feedstock for alternative fuel production. Since fossil-fuel reserves are rapidly depleting, and the price of crude petroleum is fluctuating, the need to find the importance of new fuel is growing. In addition, renewable fuels must be environmentally sustainable, inexpensive, technically appropriate, and plentiful. The chemical or lipase-catalyzed transesterification of fats and oils to produce biodiesel results in the formation of fatty acid alkyl esters, an environmentally friendly substitute liquid fuel. In addition to its green roots, it has economic as well as environmental advantages. For biodiesel production, animal fats and vegetable oils are important feedstocks. Since the conversion of edible oils into fuels is limited, demand for biodiesel made from non-edible oils is steadily rising. Researchers are therefore searching for hopeful new non-digestible oil sources that can support the formation and use of biodiesel [26].

Recently, biodiesel has become a genuine potential substitute for petroleum fuels because of a variety of desirable properties and its eco-friendly nature. However, the high cost of raw material that is used for its production is a major block to its economic viability. It has been found that biodiesel can be derived from *Eriobotrya japonica* (*E. japonica*) seeds oil by alkali-catalyzed transesterification, using the Taguchi method for enhancement of production parameters such as reaction time, catalyst amount, temperature, and alcohol-to-water molar ratio. Maximum production of biodiesel of almost 94.53% was obtained using optimum conditions including alcohol-to-water ratio 6:1, amount of catalyst 1% wt./wt., temperature 50 °C, and reaction time 2 h. The ideal conditions for obtaining 94.52% were found to be the reaction temperature. The catalyst amount (67.32 percent) had the highest contribution, followed by molar alcohol-to-oil ratio (25.51 percent). Significant fuel characteristics of *E. japonia* methyl esters formed under optimum conditions within the defined ASTM D6751 limits have been established. Biodiesel may also be considered a prospective replacement for petro-diesel [18].

Biodiesel is biodegradable and non-toxic, as opposed to petroleum diesel. The need for food competes with the utilization of edible vegetable oils for the formation of biodiesel. As long-term biodiesel sources, the continuously growing price makes biodiesel made from edible vegetable oils uneconomical. Waste vegetable oils, in addition to being a possible primary source for the formation of biodiesel, are considered to be environmental pollution, in addition to being inexpensive and easily accessible [27,28,29,30,31]. In the presence of a catalyst, transesterification of vegetable oil is carried out to generate biodiesel. The catalyst can be homogeneous, heterogeneous, enzymatic, or nanoparticles. Homogeneous catalysts are thought to be more efficient than heterogeneous counterparts due to lower mass transfer limitations and higher conversion. Because of the difficulties in separating and purifying biodiesel derived from homogeneously acid/base-catalyzed transesterification of vegetable oils, the emphasis has shifted to non-catalytic supercritical ethanol/methanol. It has been recorded that 95 percent conversion can be achieved using supercritical methanol at a reaction temperature of 350 °C, a methanol/oil molar ratio of 42, and a time of 400 s. In addition to the impact of the process variable, this study discusses and presents the benefits and drawbacks of biodiesel production processes, as well as illustrating the main data set that is currently unavailable, which would improve commercialization and economics and increase biodiesel production versus other energy sources [27,31,32,33,34,35,36,37,38].

## 4. Conclusions

The current study focuses on the preparation and properties of fatty acid methyl esters formed by base-catalyzed transesterification of *T. indica* seed oil, a potential biodiesel feedstock. The TIMEs were prepared with methanol as the acyl acceptor and NaOH as the catalyst. The Taguchi method revealed that optimum parameters affecting the transesterification of seed oil are a molar ratio of alcohol to oil of 6:1, a catalyst amount of 1.5% *w*/*w*, and reaction time of 1 h. The experiment yield of TIMEs was 93.5%, to which ‘molar ratio of alcohol to oil’ contributed the most (75.9%), followed by the amount of catalyst (20.7%). Moreover, significant fuel characteristics such as cetane number, flash point, kinematic viscosity, pour point, and cloud point were within ASTM D6751 standard limits. Based on the above findings, *T. indica* seed oil can be considered a suitable non-edible alternative feedstock for biodiesel synthesis. Biodiesel production in this way can aid in resolving concerns such as the food vs. fuels pressure on edible oil feedstock commonly used for commercial biodiesel production and the negative environmental impact of fossil-fuel use.

## Figures and Tables

**Table 1:** Parameters selected for optimization of transesterification of *T. indica* seed oil.

Parameters		Levels		
		1	2	3
a	Methanol-to-oil ratio (in moles)	3:1	6:1	9:1
b	Amount of catalyst (wt. % of oil)	0.5	1.0	1.5
c	Reaction time (min)	60	90	120

a = Methanol-to-oil ratio, b = Amount of catalyst and c = Reaction time.

**Table 2:** Design of experiment using orthogonal array with three variables at three levels (3^3^) of *T. indica* seed oil for transesterification.

	Levels and Parameters		
Experiment No.	Molar Ratio of Methanol to Oil	Catalyst Amount	Reaction Time
1	3:1	0.5	60
2	3:1	1.0	90
3	3:1	1.5	120
4	6:1	0.5	90
5	6:1	1.0	120
6	6:1	1.5	60
7	9:1	0.5	120
8	9:1	1.0	60
9	9:1	1.5	90

**Table 3:** Physicochemical properties of *T. indica* seed oil.

Property	Value
Physical State	Liquid
Color	Yellow
Density	0.840 g/cm^3^
Kinematic Viscosity	29.5 mm^2^/s
Refractive Index	1.42
FFA	1.97%
Saponification Number	202.7 mg KOH/g
Distillation Temperature Range	140–212 °C
Iodine Value	76 g I_2_ / 100 g oil

**Table 4:** Fatty acid profile of *T. indica* seed oil.

Fatty Acid	*Tamarindus indica* Seed Oil (%)	*Eriobotrya japonica* Seed Oil [18]	Rubber Seed Oil [19]
Palmitic acid (C_16:0_)	9.90	10.94	10.2
Stearic acid (C_18:0_)	2.22	2.09	8.7
Oleic acid (C_18:1_)	14.52	26.31	26.4
Linoleic acid (C_18:2_)	61.51	38.33	39.6
Linolenic acid (C_18:3_)	-	20	16.3
Eicosanoic acid (C_20:0_)	1.50	-	-
Behenic acid (C_22:0_)	3.90	-	-
Tetracosanoic acid (C_24:0_)	6.45	-	-
Total saturated fatty acid	23.97	13.03	18.9
Total unsaturated fatty acid	76.03	84.64	82.3

**Table 5:** Percentage yield of TIMEs and SNRs for the experiments designed by L_9_ orthogonal array.

Experiment No.	A	B	C	TIMEs Yield (%)			Mean Yield (%)	SNR
				Trial 1	Trial 2	Trial 3		
1	3:1	0.5	60	61.5	60.5	62	61.33	35.75
2	3:1	1.0	90	70.5	71	69	70.17	36.42
3	3:1	1.5	120	74.5	73.5	74	74.0	37.36
4	6:1	0.5	90	80.5	80	79	79.83	38.01
5	6:1	1.0	120	91.5	91	91.5	91.33	39.23
6	6:1	1.5	60	93	93.5	93	93.17	39.41
7	9:1	0.5	120	76	75.5	76	75.83	37.60
8	9:1	1.0	60	79	80	79	79.33	37.91
9	9:1	1.5	90	82	82.5	83	82.5	38.32
SNR_T_ = 37.78								

**Table 6:** Level mean SNR (SNR_L_) for different parameter levels.

Parameter		Levels		
		1	2	3
A	Molar ratio of alcohol to oil	36.51	38.88	37.94
B	Amount of catalyst (wt. % of oil)	37.12	37.85	38.36
C	Reaction time (min)	37.69	37.58	38.06

**Table 7:** Percentage contribution of variables selected for mean yield of TIME_S_.

Parameter	SS_f_	Contribution (%)
Molar ratio of alcohol to oil	2.8456	75.9
Amount of catalyst (wt. % of oil)	0.377	20.7
Reaction time (min)	0.1265	3.37

**Table 8:** The fuel properties of *T. indica* methyl esters.

Property	ASTM Test Method	Result	ASTM Limit
Kinematic viscosity @ 40 °C	D445	5.4 mm^2^/s	1.9–6.0
Acid value	D664	0.31 mg KOH/g	0.50 max
Cloud point	D2500	1 °C	-
Pour point	D97	−2 °C	-
Flash point	D93	180 °C	93 min
Cetane number	D613	47	47 min
Cu strip corrosion	D1160	1	3 max

## Data Availability

Not applicable.

## Sample Availability

Samples of the compounds are available from the authors.
